# Systems thinking and efficiency under emissions constraints: Addressing rebound effects in digital innovation and policy

**DOI:** 10.1016/j.patter.2023.100679

**Published:** 2023-02-10

**Authors:** Kelly Widdicks, Federica Lucivero, Gabrielle Samuel, Lucas Somavilla Croxatto, Marcia Tavares Smith, Carolyn Ten Holter, Mike Berners-Lee, Gordon S. Blair, Marina Jirotka, Bran Knowles, Steven Sorrell, Miriam Börjesson Rivera, Caroline Cook, Vlad C. Coroamă, Timothy J. Foxon, Jeffrey Hardy, Lorenz M. Hilty, Simon Hinterholzer, Birgit Penzenstadler

**Affiliations:** 1 Lancaster University, Lancaster, UK; 2 University of Oxford, Oxford, UK; 3 Kings College London, London, UK; 4 University College London, London, UK; 5 Small World Consulting, Cumbria, UK; 6 UK Centre for Ecology and Hydrology, Lancaster, UK; 7 University of Sussex, Brighton, UK; 8 Uppsala University, Uppsala, Sweden; 9 Baillie Gifford, Edinburgh, UK; 10 Technische Universität Berlin, Berlin, Germany; 11 Imperial College London, London, UK; 12 University of Zurich, Zürich, Switzerland; 13 Borderstep Institute, Berlin, Germany; 14 Chalmers University of Technology, Göteborg, Sweden; 15 Lappeenranta University of Technology, Lappeenranta, Finland

**Keywords:** ICT, environmental impacts, rebound effects, systems thinking, emissions constraints, digital innovation, policy

## Abstract

Innovations and efficiencies in digital technology have lately been depicted as paramount in the green transition to enable the reduction of greenhouse gas emissions, both in the information and communication technology (ICT) sector and the wider economy. This, however, fails to adequately account for rebound effects that can offset emission savings and, in the worst case, increase emissions. In this perspective, we draw on a transdisciplinary workshop with 19 experts from carbon accounting, digital sustainability research, ethics, sociology, public policy, and sustainable business to expose the challenges of addressing rebound effects in digital innovation processes and associated policy. We utilize a responsible innovation approach to uncover potential ways forward for incorporating rebound effects in these domains, concluding that addressing ICT-related rebound effects ultimately requires a shift from an ICT efficiency-centered perspective to a “systems thinking” model, which aims to understand efficiency as one solution among others that requires constraints on emissions for ICT environmental savings to be realized.

## The bigger picture

Information communication technology (ICT)’s environmental impact must be considered in digital innovation and associated policy to mitigate ICT’s climate change contribution. A proposed solution to reduce ICT emissions is by improving efficiency, yet this fails to consider rebound effects where efficiency improvements offset emissions savings or increase emissions. In this perspective, we reveal insights from a transdisciplinary workshop that identified challenges for why rebound effects are difficult to include in innovation and policy. From this, we call researchers to (1) find new ways of presenting rebound effects to digital innovators and policymakers; (2) gather cross-disciplinary evidence of ICT rebound effects; and (3) transparently analyze ICT’s environmental, societal, and economic impacts together. We also call for a systems thinking approach to addressing ICT’s environmental impacts, whereby a solution to rebound effects becomes visible: efficiencies under emission constraints.

## Graphical abstract

**Figure:**
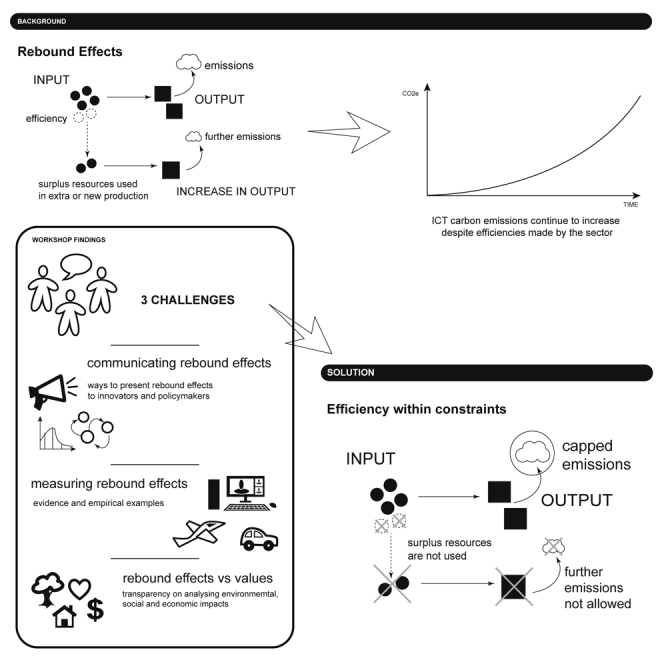

## Teaser

Rebound effects occur in the digital sector when ICT efficiency improvements offset emissions savings or increase emissions. These must be considered in digital innovation and associated policy for sustainable ICT; however, there are key challenges to this that we identify through a transdisciplinary workshop and discuss in this perspective. We call for future work to address these challenges and to take a systems thinking approach for considering ICT’s environmental impacts. From this, a solution to rebound effects becomes visible: efficiencies under emissions constraints.

## Introduction

Global greenhouse gas (GHG) emissions, including those from the information and communication technology (ICT) sector, have been rising year after year, with ICT forming 2.1%–3.9% of total global GHGs in 2020.^1^ This contribution will likely further increase with the growing range of ICT applications, accelerating demand for novel ICT (e.g., artificial intelligence [AI], Blockchain, Internet of Things, robotics), and the increasing reliance on digital technologies to support the net zero transition in other sectors (e.g., GeSI^2^). The efficiency gains ICT creates in these sectors, as well as its own, are usually viewed as central to reducing emissions worldwide because less energy is required for the same output.

This assumption ignores the behavioral and social responses as well as the adjustments to the wider economic system that typically occur when the efficiency of a process is improved. Efficiency improvements reduce the cost of the relevant good or service, which can encourage an increase in demand. There are many historic precedents for this. Jevons’ famous observation in 1865 that increasing efficiency in UK coal use in 1865 led to increases in demand^3^ led to the coining of the phrase Jevons paradox*.* More recently, increases in land transport use were seen when more efficient transportation services were introduced, such as electric trains instead of steam trains and horses.^4^^,^^5^ As these examples show, increases in demand offset emission savings and, in some cases, can lead to an overall increase in emissions (“backfire”).^6^ Emissions from the ICT industry over the last 50 years have risen from almost zero to their current level due to growing demand for digital technology despite rapid improvements in efficiency.

Across the global economy, energy efficiencies in the provision of most products and services have gone hand in hand with an average 1.8% per year growth in global carbon emissions since 1850.^7^ Emissions have continued to rise since then with little obvious deviation from that trajectory other than a small and potentially temporary dent during the COVID-19 pandemic. This does not prove that efficiency improvements lead to increased emissions, but for this not to be the case requires that emissions would have risen much quicker in the absence of efficiency gains.

Environmental rebound effects have been well categorized in the ICT-related research literature.^8^^,^^9^^,^^10^^,^^11^^,^^12^^,^^13^^,^^14^^,^^15^ Although there is no general agreed-upon taxonomy for rebound effects, most of the literature distinguishes between direct and indirect rebound effects. Direct rebound effects occur for the same good or service that had originally become more efficient and because the efficiency gains made it cheaper and thus encouraged an increase in demand. Meanwhile, indirect rebound describes a large collection of subtler and more elusive mechanisms, which also lead to an increase in consumption but of a different good or service.^16^ Indirect rebound effects include, for example, the income effect (i.e., when the monetary savings achieved from more efficient production are spent on different goods and services) or the time rebound (i.e., not energy but time is saved in the first place, time then spent on energy-intensive activities).

At the same time, sustainability and environmental impacts in general, and rebound effects in particular, are largely neglected in the design, development, or innovation processes for ICT. For example, technologists typically lack the knowledge and tools to assess environmental sustainability in software engineering practices.^17^ The full emissions life cycle and rebound effects are also overlooked in the greening of technologies such as 5G,^18^ with such “green IT” initiatives abstracting away from the social complexities that conflict with technological solutions to environmental problems.^19^ As consumption of ICT and the efficiencies it can create are placed at the heart of global efforts to address the climate crisis, there is a strong need for rebound effects to be included at the center of any digital sustainability agenda so that the climate benefits afforded by ICT efficiencies are not materially overestimated. To do so requires the development of digital technology innovation processes that contribute to the green agenda (i.e., “enablement” reducing emissions) while also mitigating the associated rebound effects. And further, in grappling with ICT’s emissions, these processes need to consider ICT’s other contributions to, and impacts on, human prosperity and the environment beyond emissions (e.g., to ecosystems and biodiversity, ICT’s use of natural resources, e-waste, etc.).

The practice of “responsible innovation” offers a useful approach to achieve this, drawing on multiple perspectives to ensure transdisciplinary expertise informs such design and development processes. Although responsible innovation can be interpreted in many ways, one especially useful definition in terms of sustainability comes from the European Union’s Responsible Research Innovation (RRI) Tools project:

RRI is a way to do research that takes a long-term perspective on the type of world in which we want to live. (www.rri-tools.eu)

This particular conceptualization of responsibility, a forward-looking, “care”-based approach to the future, highlights the sense of actively reflecting on current developments and seeking to shape them toward societal benefit. The approach most frequently seen in the UK context is drawn from the work of Stilgoe et al.,^20^ whose framework for responsible innovation consists of four dimensions: anticipation—future-based thinking to pre-empt opportunities and increase resilience; reflexivity—challenging assumptions surrounding the role of science and innovation; inclusion—supporting cohesive input from stakeholders and the public; and responsiveness—changing innovation directions based on current challenges. Responsible innovation thus actively seeks to pre-empt and respond to possible outcomes, both positive and negative, and purposefully considering and reflecting on such possible outcomes is a form of anticipatory governance.^21^ Pertinent questions include not only “what could go wrong?” but “what happens if we succeed?” The outcomes of success can be just as challenging in terms of societal impacts as those of failure—for example AirBnB’s progress from a peer-to-peer sharing app to presenting serious challenges to city planners and councils.^22^ It is the problem of “success” that can inform work on rebound effects: ICT can indeed create efficiencies—but to such good effect that consumption increases, too.

We drew on these inclusive and reflexive responsible innovation principles to design a transdisciplinary expert workshop that explored stakeholder perceptions about the nature of rebound effects, the practical challenges in accounting for rebound effects in digital technology innovation, and how these might be overcome in devising sustainable innovation processes and associated policy that meaningfully attends to wider system effects. In this perspective, we report on the key themes that emerged from the workshop. These included difficulties in communicating rebound effects, difficulties in measuring rebound effects, and the tensions between rebound effects and values. We argue that the ICT sector requires a transformation in the way it approaches efficiencies for GHG emission reductions: from unlimited increases in efficiencies and innovation to systems thinking, which accounts for efficiencies under constraints.

## Method

**Figure 1:**
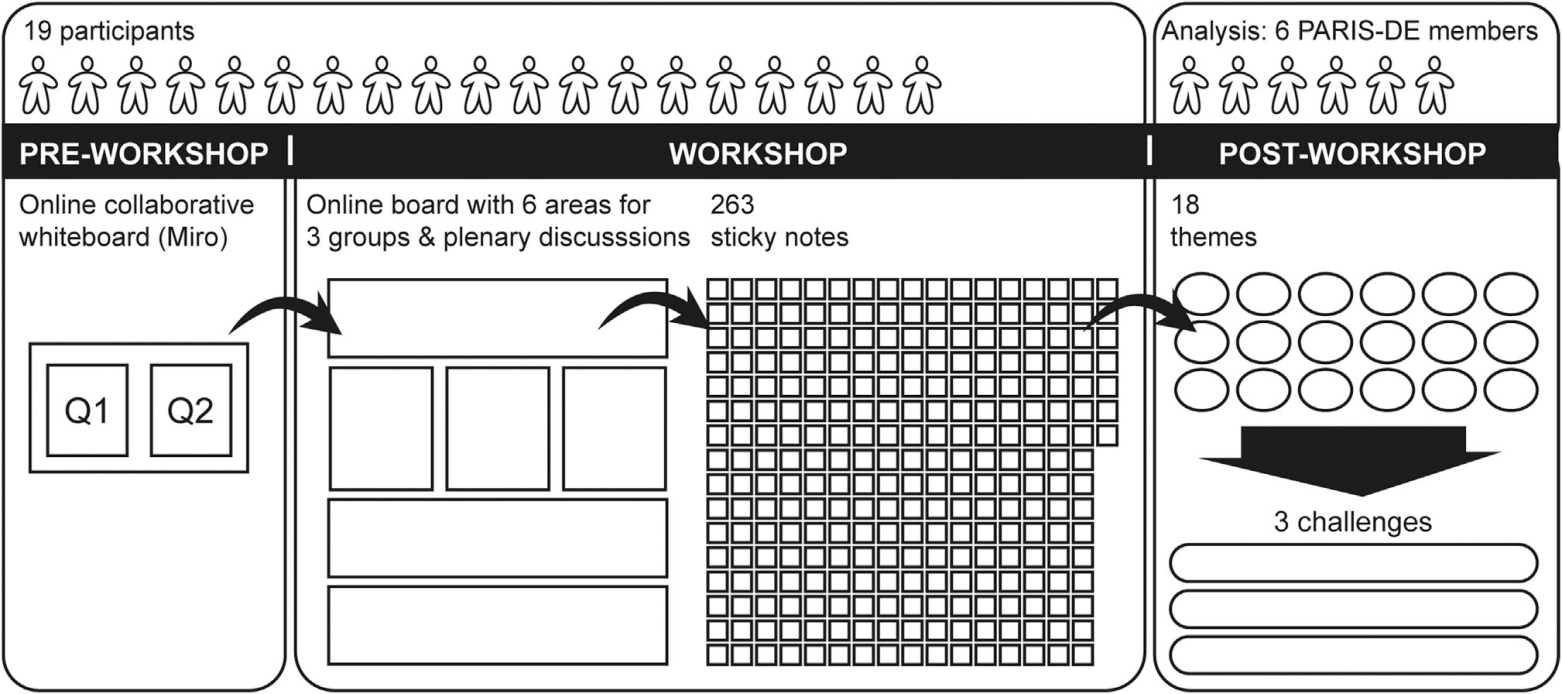
An overview of the workshop activities, participants, and analysis

To explore the complexities of rebound effects and how these can be considered in ICT innovation and associated policy, the PARIS-DE project (https://www.paris-de.org/) designed and ran a transdisciplinary 3-h online expert workshop in April 2022 that connected multiple disciplines and stakeholder expertise on this topic (Figure 1). Disciplinary backgrounds included carbon accounting, digital sustainability research, ethics, sociology, public policy, and sustainable business. Nineteen experts took part in the workshop; these experts were known to the PARIS-DE team or invited via snowballing, and while consideration was taken to gather a diverse set of participants in terms of disciplinary background and gender beyond the UK, the experts form a non-probabilistic sample from the Global North. We suggest that our findings should be complemented with a workshop formed by participants from the Global South, which we aim to conduct in our future work. The workshop was conducted via Microsoft Teams using an online collaborative Miro board. Discussions were audio recorded for note purposes only, and these recordings were deleted as soon as notes were confirmed to have been captured on the Miro board.

Prior to the workshop, participants were asked to access the Miro board and use digital notes to record their answers to the questions. This permitted the workshop organizers to gather individual experiences, perceptions, and disciplinary perspectives from the experts. The questions were as follows:

(1) In the context of your own work and experiences, what insights or examples do you have about rebound effects? For example: case studies, theoretical approaches, challenges, policy implications. This question was left open to support participants in sharing anything they deemed relevant to rebound effects and digital sustainability.
(2) What new insights are you excited to learn regarding rebound effects? This question was chosen to help identify any gaps regarding our understandings of rebound effects.

Answers on the Miro board were arranged into initial discussion points by the PARIS-DE team, which were summarized and presented at the workshop in the first activity (see below). These included the complexity of rebound effects and the difficulty in measuring or communicating them and policy and solutions or countermeasures to rebound effects, as well as specific definitions, examples, and evidence of their existence. These shaped discussions in the workshop in the second activity (see below) and, subsequently, the findings presented in this perspective (see challenges of rebound effects from ICT).

The workshop was organized into three 45-min interactive activities, each separated by 15-min breaks.

(1) Sharing perspectives. The PARIS-DE team welcomed all workshop participants, summarized the pre-workshop activity themes, and provided an open session for reflection and discussion based on these varying perspectives.
(2) Breakout discussions. With our shared perspectives as a baseline, workshop participants were divided into three breakout groups to discuss what rebound effects mean for innovation processes and public policy in relation to digital technology. Each breakout group was asked to choose a member to provide feedback for the final session, with notes from their discussions added to the Miro board.
(3) Summary reflection. Each breakout group was asked to summarize their discussions, utilizing notes on the Miro board where needed. A member of the PARIS-DE team then synthesized all group discussions into one summary, with input from all participants. The workshop concluded with a short planning session on next steps.

Following the workshop, six members of the PARIS-DE team—each with varying disciplinary perspectives from computing, design, ethics, sociology, and policy—conducted a collaborative thematic analysis of the workshop data. This involved ensuring all points from the audio recordings of the workshop were captured on the Miro board, as well as inductively identifying themes manually from the workshop notes on another Miro board during additional online meetings (see Figure 1). This exercise formed an in-depth reflection on the issues and themes that had arisen in the stakeholder group. In total, 263 digital sticky notes were created and organized into initial themes through three online sessions, forming over 7 h of collaborative analysis. These themes included definitions of rebound effects; understanding rebound effects; assessment methods; economic links; enablement; case study examples; the effect of user/people’s behavior; associated values, benefits, or positive impacts; implications (e.g., design strategies, methodological solutions); responsibility; sufficiency; policy; incentives; social and environmental justice; constraints; and communicating and framing rebound effects. Additional rounds of thematic analysis led to three overarching themes each with emerging challenges (see challenges of rebound effects from ICT), as well as underpinning themes for discussion (see systems thinking in digital innovation and policy), which together form the basis of this perspective. Thematic analysis on the Miro board as well as the draft paper were shared with all participants, who were invited to contribute to the further development and refinement of the insights through this perspective. All participants are therefore authors of the perspective and have provided consent for their discussions to be published.

## Challenges of rebound effects from ICT

In this section, we provide an overview of the core themes that emerged from the workshop discussions and identify a key challenge that emerges from each of these.

### Communicating rebounds

Workshop participants emphasized the need to take care when communicating issues associated with rebound effects in efficiency-based solutions given how language can convey positive or negative meanings and/or feelings depending on the terms and analogies used. Language can—depending on context—be understood and used in different ways to fit different narrative purposes: a more “efficient” system is well known to support increases in production and profit, yet simultaneously, a more “efficient” system is depicted as one that decreases emissions. Danger lies in conflating these two conflicting meanings.

Participants were concerned that the language associated with rebound effects may promote a defensive response, ultimately discouraging digital innovators from engaging with the topic and creating more successful solutions. They were also concerned about how certain language could lead to a sense of doom, with digital innovators’ and policymakers’ efforts potentially feeling useless. As sustainability is often placed in opposition to growth in this debate, participants expressed that these messages can even push some digital innovators and policymakers away from engaging with rebound effects when their primary job is to deliver economic growth and prosperity to businesses and populations.

Participants discussed whether the use of a different communication strategy would promote engagement of innovators and policymakers in discussions about rebound effects: for example, by using the language of digital sufficiency.^23^ Moving forward, one workshop participant suggested that drawing examples of communication strategies from other sectors, such as health and education, could help the ICT sector communicate with policymakers and influence change in policy. Utilizing imagery, diagrams, and visualizations to communicate rebound effects for easier comprehension by digital innovators and policymakers was also considered. Such images were viewed as a way to help communicate a complex issue such as rebound effects in an accessible way, highlighting relationships and interconnections between different parts of the system.^24^^,^^25^

There was a clear consensus that current communication strategies were not working, and new ways of presenting the issue are needed to ensure they are considered in ICT innovation and associated policy.

Challenge: To find new ways of presenting rebound effects to innovators and policymakers in the digital sector to ensure they are addressed in innovation and associated policy.

### Measuring rebounds

Given that ICT pervades multiple activities and sectors worldwide, the total of all environmental rebound effects (direct and indirect) includes an infinite number of possible mechanisms rippling throughout the global economy and involving complex interactions between every sector. Consequently, workshop participants emphasized that this makes environmental rebound effects extremely difficult to quantify. As these rebound effects also occur in the future, participants discussed how rebound estimates require the use of scenarios rather than measurement, adding further uncertainty.

Studies that do attempt to quantify rebound effects introduce boundary constraints for what impacts they do consider, often adding together potential individual rebound effects in order to calculate a rough overall estimate. Yet, attempts to quantify the overall rebound effect of one efficiency improvement by adding up the effect of each rebound pathway result in an underestimate of the total effect due to truncation of myriad mechanisms at play. While some evidence of total rebound effect can be found by correlating efficiency gains with total emissions, even here, the exercise is complicated by the interactions between different sectors of the economy, and ultimately it is not possible to consider the effect of one efficiency improvement in isolation from the others that are simultaneously taking place.

Workshop participants expressed the need for new methods to anticipate rebound effects. While rebound quantification could provide clear guidance for decision-making around ICT’s design and environmental impacts, more qualitative assessments were simultaneously called for, as waiting for concrete and cohesive evidence from comprehensive quantitative models and measurements would be somewhat futile. This is not only because of the urgency of the climate crisis but also because considerable uncertainty will remain, and we need to be able to digitally innovate and govern within these realms of uncertainty.^26^^,^^27^ Specific solutions included trialing methods such as the anticipatory governance approaches described above^28^ or futures thinking and scenarios in design and innovation^29^^,^^30^^,^^31^^,^^32^ for identifying and considering potential rebound effects.

With data on ICT’s environmental impacts being scarce, building a collection of rebound examples and case studies—detailing how they happen and why they occur—and then drawing on these in ICT innovation processes to explore solutions was viewed as a necessary requirement by workshop participants moving forward. One discussed example was the role of ICT—and specifically video conferencing—on the travel industry. While regional hubs connected via video conferencing can significantly reduce the emissions of a conference,^33^ there is evidence that the demand for video data, and flights more generally, have increased year by year alongside ICT and aviation emissions (apart from the COVID-19 pandemic), meaning video does not substitute all flying.^1^ Moreover, teleworkers have been found to travel more for business each week than non-teleworkers.^34^ Accumulating more examples such as these, as well as others in the literature, would provide useful evidence on ICT’s impacts and how and when rebound effects occur, particularly in relation to the emissions of other sectors.

Despite the suggestions for a collection of rebound examples and case studies, concerns remained among workshop participants that adopting quantitative and qualitative tools for evidencing rebound effects alongside each other requires embracing the use of mixed methods data. Specifically, drawing on well-established controversies in the social sciences, participants noted that incorporating qualitative data would make rebound effects difficult to evaluate in line with available quantitative data, meaning that evaluating rebounds could concomitantly make measuring them in digital innovation and policy even more complex.

Without reaching a solution to these complexities, there was a clear demand from workshop participants to address these issues—specifically trialing different methodological approaches and bringing together rebound effect examples in practice to help the digital sector in considering and addressing rebound effects.

Challenge: To generate and collate cross-disciplinary evidence and empirical examples of rebound effects in ICT.

### Tensions between rebounds and values

Not only are direct environmental rebound effects difficult to identify, confine, and measure, but workshop participants discussed how they must also be considered in tandem with social and economic dynamics. These could be perceived either positively or negatively, depending on the discipline, leading to inherent tensions between the benefits and drawbacks of different rebound effects. Economic growth that emerges from efficiency gains, and which contributes to increased employment, prosperity, and/or increased well-being, was a core example provided by workshop participants. Most economists thus view economic growth as a positive rebound that emerges from increased efficiency. On the other hand, environmentalists are concerned that such economic growth can (and will) lead to negative environmental impacts, and so the evaluation of efficiency improvements must also consider limits to material production and consumption, environmental degradation, and social inequalities.^35^^,^^36^

In trying to disentangle some of these issues, participants questioned how best to conceptualize and actualize the relationship between economic, social, and environmental imperatives, each of which potentially brings a range of value-laden assumptions and divergent values.^37^^,^^38^ One approach is to unpack and rethink assumptions engrained in our contemporary value systems. The need for economic growth was one such assumption interrogated by participants at the workshop. The well-established alternative economic models that consider notions associated with whom economic growth is intended for (“growth for whom; whose needs are being met?”) were viewed as vital in this space (e.g., Doughnut Economics^39^). Discussions also emphasized the need to ensure that multiple dimensions of sustainable development that go beyond GHG emissions are considered.^40^^,^^41^ For example, social well-being and the need for development worldwide need to be taken into account, including, but not limited to, issues of war, energy security, food security, access to employment, and cost of living.

In summary, and for rebound effects to be addressed in digital innovation and associated policy, experts agreed that all rebound effects (direct or indirect), as well as social and economic dynamics, need to be considered in tandem, with transparency of competing values.

Challenge: To transparently and thoroughly analyze ICT’s environmental, societal, and economic impacts to account for digital technology’s rebound effects.

## Systems thinking in digital innovation and policy

The difficulty of communicating the mechanisms of rebound effects to non-experts, the challenges in measuring those effects, and their conflicts with differing values are all likely reasons our transdisciplinary workshop surfaced for why environmental rebound effects are often not considered in practice, and why it is challenging to devise policy to address them. What ultimately underpins these challenges is that assessment of ICT requires balancing known impacts—such as economic growth, unemployment, and cost of living—with unquantifiable risks and uncertainties associated with social and environmental impacts. We argue that these difficulties with considering rebound effects are compounded by a current socio-political context that promotes techno-solutionist and efficiency-centric perspectives for how digital technology impacts the economy, societies, and the environment. Until we address this context, we are unlikely to get traction in addressing the challenges presented.

Techno-solutionism^42^^,^^43^^,^^44^ is the idea that innovations are the only solutions to global challenges and will linearly lead to positive social, economic, and environmental outcomes.^2^^,^^45^ This leads to a resultant focus on digital technology to deliver reductions in global emissions and environmental impacts, which it has so far attempted to provide through efficiency gains. Yet, as we have emphasized in this perspective, efficiency on its own cannot be relied upon to reduce environmental impact. Instead, because of rebound effects, for efficiency improvements to be environmentally beneficial, they must be accompanied by a constraint on emissions. Rebound effects thus do not fit in the techno-solution narrative, as they expose the complexities of human behavior and socio-technical interactions. In fact, rebound effects challenge the simplistic causal link between innovation and positive impacts in techno-solutionism, which takes a narrow view of digital technology and sustainability, without considering the bigger picture and accounting for all these interrelated impacts.

To account for this, systems thinking is required. Systems thinking is an approach that considers complex problems from a holistic perspective across time, taking into account the relationships and dynamics among the components of a system.^46^^,^^47^^,^^48^ Meadows and Wright^46^ refer to systems thinking as a “different way of seeing and thinking,” one whereby a “system is more than the sum of its parts” and where emphasis is placed on interactions and feedback loops, resulting in emergent and often unexpected behaviors and surprises. This differs from analytical approaches that study complex issues by reducing systems into smaller parts. Systems thinking is a well-established area of study, having been applied in fields such as engineering, management, and computer science, as well as sustainability.^49^^,^^50^ Reasons for embracing this more concretely for sustainable ICT innovation and associated policy are 2-fold: systems thinking exposes the flaws of techno-solutionist, efficiency-driven narratives through making rebound effects visible, and systems thinking enables efficiencies to support environmental sustainability through the use of emissions constraints. This is because systems thinking requires a broadening of scope for what is important and what needs to be accounted for when considering the impacts of the ICT sector. With this widening, efficiency is only one solution among others that are needed to address digital technology’s environmental impacts—both in and beyond the ICT sector.

Specifically, a broader system thinking approach implies that efficiency gains can bring about negative impacts on environmental and social dimensions, and if they do, then it becomes reasonable to rethink efficiencies as a sole solution. Instead, ensuring efficiencies under constraints on GHG emissions forms a completely justifiable and appropriate response. In this sense, constraints to the additional outputs from efficiency can be seen not as a limitation to economic growth but as fostering environmental sustainability or social sustainability. As we fall deeper into the climate crisis, it has never been more important to foster environmental and social sustainability, and this solid, sustainable foundation is required for any economic endeavor, e.g., productivity through efficiency, to prosper.

**Figure 2:**
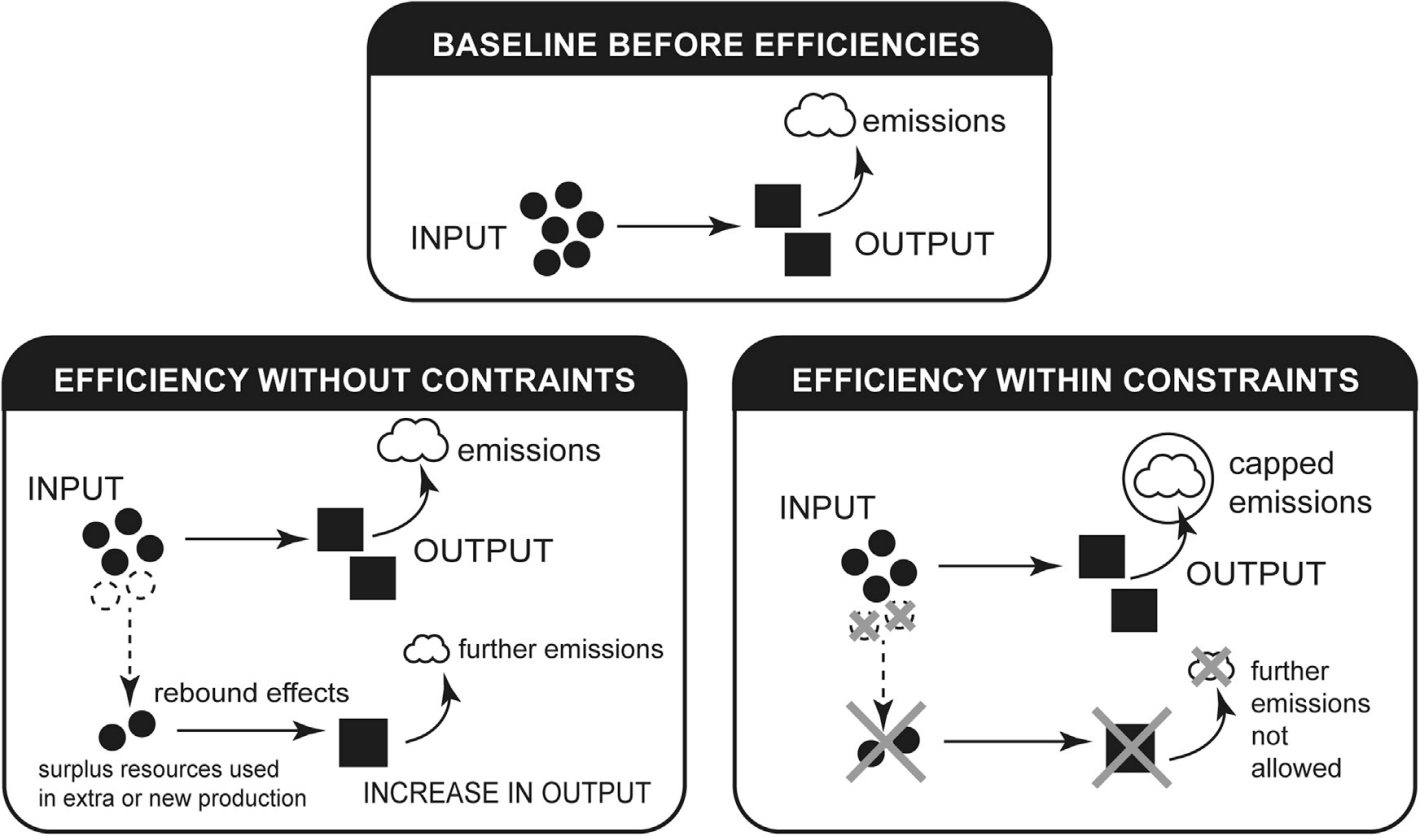
A visualization of rebound effects from efficiency and efficiency under emissions constraints

Furthermore, systems thinking changes the role of efficiency as a solution. When constraints are placed on digital technology and its environmental impacts—such as through the use of caps on GHG emissions—the dynamics of growth from efficiency would be changed completely. In this scenario, the rebound effect would be eliminated because emissions would be fixed at the level of the GHG constraint, and efficiency gains would become the sole means by which output growth would be possible. We therefore emphasize that efficiency gains rely upon GHG emission constraints to make emission reductions possible, and thus efficiencies under constraints are the only true efficiencies for emission reductions (Figure 2)*.* Moreover, with systems thinking, efficiencies under constraints are not antagonists of growth and innovation but rather provide the friction needed to drive effective progress in innovation and support wider opportunities for good for our environment, societies, and economy. Constraints therefore inspire creativity in digital innovation and associated policy to fully support the green transition. While we have focused on GHG constraints in this first instance, it is worth pointing out further constraints (e.g., on material depletion) will also be relevant for sustainable digital technology innovation to thrive and, especially, to provide a collective approach to address interconnecting rebound effects.

In summary, rebound effects represent clear challenges that the digital sector needs to address in digital innovation processes and associated policy. Systems thinking offers a sector-wide perspective change that can account for such challenges, enabling an understanding that efficiencies are one solution among others that need constraints to offer emissions savings. We urgently need to embrace this approach for digital sustainability to be realized and suggest that transdisciplinary and responsible innovation research, like this workshop, is one prerequisite to embracing systems thinking and its broader lens in order to consider the varying disciplines and stakeholder views that surface the systems and tensions between them. The economic opportunity for the ICT sector offered by embracing the significance of rebound effects and the consequent requirement for a global GHG constraints is that under those constraints, efficiency improvements, including those offered by ICT to the whole economy, will become more valued than ever as the only means by which output growth can be possible.

## Conclusion

In this perspective, we have drawn on transdisciplinary analysis of an expert workshop to explore the issue of environmental rebound effects that emerge from ICT. We have summarized key challenges surrounding why rebound effects are difficult to include and assess within digital technology innovation processes and associated policy, specifically regarding communicating rebound effects, measuring rebound effects, and the tensions between rebound effects and values. Our call for action for researchers moving forward is to (1) find new ways of presenting rebound effects to innovators and policymakers in the digital sector to ensure they are addressed in innovation and associated policy; (2) generate and collate cross-disciplinary evidence and empirical examples of rebound effects in ICT; and (3) transparently and thoroughly analyze ICT’s environmental, societal, and economic impacts in tandem to fully account for digital technology’s rebound effects. Furthermore, we recognize that these challenges relate to limitations in current techno-solutionist and efficiency-centric perspectives for how digital technology impacts the economy, societies, and the environment. Systems thinking exposes the flaws of these perspectives: efficiencies under GHG emission constraints are the only way in which efficiencies for true emission reductions can be realized. This urgently required change in approach, underpinned by transdisciplinary and responsible innovation research, is what will enable an ICT sector-wide drive for digital technology’s negative rebound effects to be assessed and overcome—enabling improvements in digital innovation for the good of our environment, societies, and economy.
